# On the Shallow Processing (Dis)Advantage: Grammar and Economy

**DOI:** 10.3389/fpsyg.2016.00082

**Published:** 2016-02-10

**Authors:** Arnout Koornneef, Eric Reuland

**Affiliations:** ^1^Brain and Education Lab, Institute for Education and Child Studies, Leiden UniversityLeiden, Netherlands; ^2^Utrecht Institute of Linguistics OTSUtrecht, Netherlands

**Keywords:** anaphoric dependencies, good-enough processing, variable binding, coreference, (reflexive) pronouns, economy hierarchy

## Abstract

In the psycholinguistic literature it has been proposed that readers and listeners often adopt a “good-enough” processing strategy in which a “shallow” representation of an utterance driven by (top-down) extra-grammatical processes has a processing advantage over a “deep” (bottom-up) grammatically-driven representation of that same utterance. In the current contribution we claim, both on theoretical and experimental grounds, that this proposal is overly simplistic. Most importantly, in the domain of anaphora there is now an accumulating body of evidence showing that the anaphoric dependencies between (reflexive) pronominals and their antecedents are subject to an economy hierarchy. In this economy hierarchy, deriving anaphoric dependencies by deep—grammatical—operations requires less processing costs than doing so by shallow—extra-grammatical—operations. In addition, in case of ambiguity when both a shallow and a deep derivation are available to the parser, the latter is actually preferred. This, we argue, contradicts the basic assumptions of the shallow–deep dichotomy and, hence, a rethinking of the good-enough processing framework is warranted.

## Introduction

The marriage between linguistic theory and experimental psycholinguistics is a tumultuous one. On the one hand the one cannot live without the other, on the other hand, their relationship is characterized by frequent quarrels and misunderstandings. The tension is nicely shown by the following two quotes on “deep” vs. “shallow” processing. One is from Marantz (class lectures, 2000):

(1) *Deep processing* (our label, borrowed from the literature)

“The split between linguistics and psycholinguistics in the 1970's has been interpreted as being a retreat by linguists from the notion that every operation of the grammar is a mental operation that a speaker must perform in speaking and understanding language. But, putting history aside for the moment, we as linguists cannot take the position that there is another way to construct mental representations of sentences other than the machinery of grammar….There is no retreat from the strictest possible interpretation of grammatical operations as the only way to construct linguistic representations.”

The other position is aptly illustrated by a quote from Ferreira (2003):

(2) *Shallow processing*

“The results …. suggest that a comprehensive theory of language comprehension must assume that simple processing heuristics are used during processing in addition to (and perhaps sometimes instead of) syntactic algorithms. Moreover, the experiments support the idea that language processing is often based on shallow processing, yielding a merely “good enough” rather than a detailed linguistic representation of an utterance's meaning.”

Over the last decade, Ferreira's and related positions prompted a substantial line of research examining the driving forces in language processing. The broad scope of Ferreira's claim becomes particularly clear in a recent elaboration of the shallow, or “good-enough,” processing position in Karimi and Ferreira (2015). That is, from their comprehensive overview of the literature and their implementation of a general “online cognitive equilibrium” model of language processing, one must conclude that they intend the good-enough processing position to hold generally across linguistic domains, arguing that “algorithmic procedures for sentence processing are not only too costly but sometimes outright unnecessary.”

Since the shallow processing position has become an influential one, it deserves careful scrutiny. Yet, if we want to fully understand it, we are facing the fact that the mechanisms for shallow processing have not been formulated explicitly: it remains unclear how, precisely, they do their work. For instance, in order to fully understand the quote in (2) it is important to have a clear understanding of what counts as a heuristic. However, as Karimi and Ferreira state themselves, “the nature of the simple rules that guide heuristic processing is unclear.” They do, however, provide the following helpful characterization: “We believe that this (= heuristic, K&R) processing relies more heavily on top-down information from semantic memory, whereas algorithmic processing seems to rely more heavily on linguistic knowledge to derive meaning in a bottom-up way, by organizing and combining the unfolding input using well-defined, successive linguistic rules. “It this ‘top-down’ vs. ‘bottom-up’ characterization of the relevant contrast, we will rely on in our discussions in the current contribution.

Furthermore, we would like to submit that if one wants to argue that in a particular situation people only assign a shallow interpretation, there is no escape from the requirement to make precise what this interpretation is, and which processes are involved in its derivation. Also in this respect the good-enough approach leaves some fundamental questions open.

### The nature of good-enough representations in language processing

To illustrate the issues raised above further, let's think more carefully about the basic question of what counts as a good-enough representation. Note, that this question relates to rather fundamental questions about meaning representation. But, for the purpose of the present contribution we will try to stay as concrete as possible. Consider, then, the utterance in (3):

(3) The girl pushed the boy.

We would take it that in order to be good enough the utterance should be interpreted as representing a pushing relation rather than a kissing relation or an injuring relation. But, suppose we know that the pushing resulted in an injury, would a representation as an injuring relation still not be good enough? Perhaps it is, perhaps it isn't. In any case, it seems to us that a representation in which the boy does the pushing and the girl is pushed would certainly not count as good enough.

Now consider a case with quantifiers as in (4):

(4) Some girl pushed every boy.

Which scopal relation should count as good enough? Suppose *every boy* scoping over *some girl* is intended (with each boy being pushed by a different girl), is then the alternative with *some girl* scoping over *every boy* (that is, all the boys are being pushed by the same girl) still good enough? It seems, then, that the notion “good enough” in isolation is problematic. And in fact this is already illustrated in Ferreira's (2003) discussion. As she shows, in a remarkable number of cases participants assign a wrong interpretation to sentences. Thus, the question to ask is what representation, given limitations on attention and processing resources, will have to make do for a particular hearer in a particular situation, and how it is derived.

One area that provides a simple illustration of the different perspectives and the problems associated with the notion good enough is the interpretation of reversible passives as in (5) (Grodzinsky, 1995; Ferreira, 2003). As is well-known, children, agrammatic aphasics, but also certain typical speakers with no known deficit (Ferreira, 2003), show problems interpreting reversible passives^1^. For instance, agrammatic aphasics may show above chance performance on the active (5a), but only chance performance on the passive (5b), allowing (5c) as a possible interpretation.

(5) a. The girl pushes the boy. (*Above chance performance*).
b. The boy is pushed by the girl. (*Chance performance*).
c. The boy pushes the girl.

One may then hypothesize (as did Grodzinsky) that agrammatic aphasics cannot link the surface position of *the boy* to the object position in which it is assigned its semantic role. As such, *the boy* cannot receive a theme role. Or, in a less explicit manner, hypothesize, as did Ferreira (2003) in a study of typical participants, that there is a cost in following the grammatical algorithms. Consequently, in this view, the participants in these tests resort to an extra-grammatical interpretation strategy, based on the idea that there is a hierarchy of semantic roles and that the *agent* role is the most prominent role in this hierarchy^2^.

(6) Assign the *agent* role to the leftmost NP of the clause as a default role.

This simple rule of *agent first* is indeed a good example of a top-down heuristic involving an extra-grammatical principle. Participants use this strategy to assign a role to *the boy* and interpret (5b) as (5c). Thus, crucially, in such a case the meaning representation arrived at is certainly not good enough. In fact it is not good at all. But it is *the* representation the participant arrives at and for him or her has to make do.

From these and similar results Ferreira (2003; see also e.g., Karimi and Ferreira, 2015) concludes that the field of language comprehension should adopt an approach similar to that taken in the Fast and Frugal Heuristics models (Gigerenzer et al., 1999; Gigerenzer, 2000), who take the position that “Models of rational choice which assume ‘unbounded rationality’ are unrealistic because the computations that are assumed to take place are often far too burdensome for real creatures operating in demanding environments.” In the context of our present discussion, this would imply that a complete syntactic parse of a sentence—i.e., demanding the application of a wide range of grammatical computations—often yields a situation that is too burdensome for real creatures.

A crucial assumption is, then, that shallow processing—by avoiding a full syntactic parse and applying extra-grammatical heuristics instead—is cheaper than deep processing—which is based on the application of all available syntactic algorithms. This assumption clearly embodies an empirical claim. Let's call this assumption the *shallow advantage*. A second assumption, crucial for a fast and frugal heuristic to be viable at all is that using shallow strategies can in principle yield a similar interpretive result as grammatical computations—at least roughly so, even if speakers don't always do so. That is, even if such representations may be “incomplete,” “lacking in detail,” “sketchy,” or “imprecise,” they have to be good enough to be used (cf. Karimi and Ferreira, 2015). Let's refer to this as the *shallow equivalence* assumption; a strategy that can only lead to representations that for principled reasons fail to be at least moderately equivalent to what would have been derived by grammatical computations in a particular domain (i.e., a representation that could *never* be good-enough), may be frugal, but not very fruitful for the creatures using it. It is the aim of this contribution to critically assess these assumptions, which so far received too little attention in the literature from this perspective.

In the latter sense our goals are on a par with those of Karimi and Ferreira (2015), who in their recent proposal elaborated on the core assumptions of the good-enough processing framework (e.g., Ferreira, 2003; Ferreira and Patson, 2007). They put forward two fundamental processing principles that, in fact, closely mirror the two assumptions formulated above. More specifically, Karimi and Ferreira specified that “the reason why sometimes only fast and frugal heuristics rather than deep and time-consuming algorithms are applied during comprehension could be because heuristics offer a faster route to equilibrium (Principle 1). Similarly, the reason why the system is sometimes satisfied with a good-enough representation and does not exert the extra effort to engage in deeper processing could be because heuristics often provides enough equilibrium for the system, causing it to stay in that state for as long as possible…(Principle 2)” (pp. 6). Furthermore, following Kuperberg's (2007) syntactic-semantic model, Karimi and Ferreira claim that the algorithmic route of their implementation of the good-enough approach is syntactic in nature. The alternative route, on the other hand, relies more heavily on top-down information from semantic memory, and is capable of generating more global meaning representations of a sentence (intrasentential) or discourse (intersentential)^3^.

Hence, Karimi and Ferreira's Principle 1 is identical to the shallow advantage assumption. Moreover, even though their Principle 2 is perhaps formulated less specifically than the equivalence assumption we ascribe to the good-enough position, Principle 2 reflects a similar core idea. That is, during the initial stages of processing there should be a perceived—or at least anticipated—equivalence between the output of the heuristics and algorithmic routes—after all, why should a creature be bothered with the construction of a mental representation that he or she knows will not be a reasonable reflection of the associated linguistic input?

To further substantiate their claims, Karimi and Ferreira (2015) present a comprehensive overview of studies that, in their opinion, are best explained by adopting a fast and frugal approach to language processing. These studies examined shallow linguistic processing for a wide range of different phenomena, such as the Moses Illusion, local syntactic ambiguities in garden-path sentences, quantifier scope ambiguities, erroneous interpretations of syntactically complex sentences, and the resolution processes of referring expressions (for references and more discussion, see Karimi and Ferreira, 2015).

As becomes clear from the discussion of Karimi and Ferreira (and as pointed out to us by one reviewer), a problem that arises if we set out to evaluate the shallow processing position is that the term “shallow processing” (originally due to Carter, 1985) is being used to refer to two different types of “shallowness” that must be kept apart—although they are not entirely unrelated. One involves the *top down* use of information from semantic memory, as briefly mentioned above. The other involves what one may call *reduced processing*.

That is, in some of the processing literature (for instance Stewart et al., 2007), and also some of the cases discussed by Karimi and Ferreira, shallow processing comes down to simply not fully processing part of the input—or at least delaying its integration (cf. Von der Malsburg and Vasishth, 2013). As Stewart et al. argue, in processing an input like *Paul*_*i*_ *lent Rick*_*j*_ *the CD before he*_*i*∕*j*_ *left for the holidays* the processor my simply disregard the temporal clause initially, and only yield a representation for Paul lending the CD to Rick. This type of shallow processing does not involve extra-grammatical heuristics. There is no top-down use of information coming from semantic memory and, in fact, it is compatible with deep processing using standard grammatical algorithms of whatever has been admitted to the processing buffer. For want of a better term, we will refer to it as “shallow-by-reduction” or *shallow-R*, in order to avoid confusion.

Shallow-R processing in the Stewart et al.'s sense (i.e., as partial non-processing) is not what we primarily address in this contribution—although it still raises non-trivial questions about the representations that are being derived. Instead, we will be focusing on claims about the type of shallow processing that explicitly involves the use of top-down information—including the use of extra-grammatical *heuristics*. We will refer to this notion of shallowness as “shallow-by-top-down,” briefly *shallow-TD*.

This brings us to our main concern with these latter type of heuristics, which is actually three-fold, and can be summarized as follows: (1) it is unclear how they actually do the job they are taken to perform; (2) it is unclear whether they are necessary at all; (3) it is unclear—if they exist—why/whether they would be cheaper than the use of syntactic algorithms. We will start the discussion of these concerns on the basis of the *agent first* heuristic in the (shallow) interpretation of passive sentences—i.e., before moving on to anaphoric dependencies, which will be the main test case in the current contribution for the shallow-by-top–down position.

### The agent first heuristic in passive sentences

Linguistic theory moves forward at a considerable pace. Consequently, considerations from the past need no longer apply to the current state of affairs. For instance, if it is claimed, after Slobin (1966), that “nonreversible sentences can be understood by going directly to the semantic roles without an intervening syntactic structure,” we can easily see this is overly simplistic. As we now know, thematic role assignment is not just a matter of an argument “encountering” a predicate—containing an empty slot—in the mental working buffer and “filling the hole.” Rather, the process involved minimally depends on verb and role type as shown for the contrasts between the processing of different types of intransitive verbs demonstrated in Koring et al. (2012).

Thus, even simple intransitive predicates have more internal structure than meets the eye, and this carries over to our initial example of passives. It is important to see that—in order for there to be a meaning representation at all—*the boy* in (5b) must be assigned a position to be formally identified as a subject (checking agreement, and/or case), that is, to function as an argument of the verb and its associated functional material. Given that under this construal it receives—mistakenly—the agent role, it must be able to semantically integrate with the verb in this capacity. Furthermore, *the girl* must be construed as the object and interpreted accordingly as bearing the theme role associated with this position. There is no escape from the assumption that in assigning this interpretation to the sentence, the processor has to treat the passive verb form as the active entry to which it is lexically related (Reinhart, 2002; Reinhart and Siloni, 2005). This it can only do if it *disregards* function words, such as *by* and *is*, and morphology like—*ed*. Thus, when (5b) is in fact interpreted as (5c), the “active” computation still needs to take place, which is not necessarily shallow at all. Or to put it bluntly, also *deriving a “wrong” interpretation requires explicit computations unless one advocates resorting to magic*.

But there is a further question. Namely, is an auxiliary, heuristics-based, interpretation strategy in fact necessary in this case? Recall that (5b) can only be interpreted as (5c) if the processor disregards the relevant functional elements. But note, that if it does so, the active interpretation is the only one that can be assigned. So in fact, no recourse to auxiliary strategies is needed. It is enough to assume that under certain conditions some functional elements—here, those necessary for a passive construction—will not enter the buffer of the processing system, and the processor simply works with what is has. From the perspective of Marantz's thesis in (1), then, one may assume that in order to interpret (5b) as (5c) a sufficiently articulate structure will be projected and interpreted by the rules the grammar contains. Projecting a structure that ignores the functional material that is present to license the passive interpretation (e.g., since it does not fit in the buffer due to cognitive overload, time pressure etc.), and subsequently using the active base form of the verb, will be quite enough to derive the interpretation observed. Hence, the most parsimonious assumption is that, at least in this domain, no extra-grammatical heuristics—other than disregarding functional elements—are involved at all. In short, here, shallow-TD reduces to shallow-R^4^.

Of course, one may argue that at least in some cases utterances are interpreted by truly shallow processing. In case of high running emotions people may focus on one or two words in an utterance, completely ignoring any nuance and complexity the utterance may carry. And it is true that so far little is known about the syntax and semantics of exclamations. On the other hand, there is a growing literature on headlines and other similarly reduced linguistic expressions, which shows that these are far from arbitrary, and reflect an articulate linguistic structure underneath (De Lange, 2008). Thus, even if emotions highly limit the amount of items that are admitted into the processing system's buffer, this does not imply that whatever is admitted into the system is not subsequently structured and processed with grammatical means.

### The current contribution

The discussion on passives as presented above nicely introduces the assumptions underlying the fast and frugal heuristics model, in particular the assumption that a full syntactic parse is complex, and hence, often more costly than the extra-grammatical strategies the lazy language user has at his disposition—what we refer to as the shallow advantage. It also becomes clear that the interpretation of passives is perhaps not the best domain to further evaluate the issue of *top-down* vs. *bottom-up* strategies—since shallow-TD can be reduced to shallow-R.

In the present contribution we will focus on the domain of anaphoric dependencies instead. The underlying reason is twofold. First, in their recent overview of the literature Karimi and Ferreira (2015) explicitly state that an important case of shallow processing in discourse is reference processing, a topic that in their opinion has not received enough attention in the good-enough literature. Second, and more importantly, we will argue that the by now firmly established linguistic theories on anaphoric dependencies allow us to more directly compare shallow and deep processing. Or to frame it more in terms of a good-enough approach (and Karimi and Ferreira's remark on the cost of algorithmic procedures for sentence processing): since grammatical computations and heuristic top-down principles are taken to compete, the well-defined grammatical (deep/bottom-up) and extra-grammatical (shallow/top-down) processing mechanisms of anaphoric dependencies present the perfect testing ground to critically assess whether grammatical computations are indeed “too cumbersome for real creatures”—i.e., as compared to their extra-grammatical alternatives.

In the following sections, we will argue that they are not. That is, we will argue—primarily on theoretical grounds—that the *shallow equivalence* assumption does not hold, at least not in this specific domain (Section *The equivalence assumption for shallow and deep anaphoric dependencies*). In addition, the *shallow-TD advantage* assumption has been evaluated in several experimental studies and, as we will demonstrate, shown to be false in the domain of anaphoric dependencies (Sections *The shallow-TD advantage assumption: Preparing the ground* and *The shallow-TD advantage assumption: The issue of economy*). To us, it seems that this provides enough reason to be skeptical about the aforementioned assumptions and, hence, this particular good-enough implementation of the heuristic/top-down approach to language processing.

## The equivalence assumption for shallow and deep anaphoric dependencies

It is a fundamental property of language in its relation to the world around us—and its mental representation—that different nominal expressions may receive the same value. Although nothing forces it—i.e., putting pragmatics aside for the moment—also nothing prevents that *the old baron* and *the driver* in the following sentence are used to refer to the same person.

(7) The old baron was crossing the bridge in a ramshackle carriage. The driver was visibly tired.

In this process of valuation, a linguistic expression is assigned a value from an extra-linguistic domain. Or more specifically, which value it receives is not grammatically determined. This provides a nice case of a potentially shallow-TD operation: Take an expression and assign it a value; prima facie no deep grammatical computations involved, and neither much searching if the referent is prominent in the context (in any case not more than general heuristics may be expected to require). Perhaps Karimi and Ferreira (2015) most clearly articulated this idea, since they state that “the processing of unambiguous referring expressions is facilitated because the comprehension system quickly reaches equilibrium by establishing the referential link between the referring expression and the antecedent through a simple, quick, and heuristics-based coindexation process, leading to little if any processing difficulty.”

Whereas in sentence (7) we are dealing with two lexical noun phrases, the same option is available for pronominals, as in (8).

(8) *This soldier* has a gun. Will *he* attack?

*He* in (8) can be interpreted as the same individual as *this soldier*. However, this option is not available for all nominal expressions. The mini-discourse in (9) is infelicitous:

(9) *No soldier* has a gun. ^*^Will *he* attack?

This is due to the fact that *he* and *no soldier* cannot be co-valued. *No soldier* is quantificational, and does not introduce an entity *he* can refer to (an observation leading to the canonical distinction between binding and coreference in Heim, 1982; Reinhart, 1983, and subsequent work; see also Partee, 1978; Bosch, 1980, 1983). This makes anaphoric reference such as in (8) impossible. The same holds true of expressions like *every soldier*. Note that these well-known examples have important implications for how one should interpret the notion “discourse entity.” That is, possible discourse antecedents are as diverse as soldiers, water, beauty, head-aches, dissatisfaction, etc. In addition to these nominal expressions, also sentences, verb phrases, prepositional phrases, adjective phrases, and tenses, admit anaphoric relations. Thus, the notion discourse entity must be broad enough to capture all these cases of anaphora, yet be restrictive enough to separate them from quantificational cases such as *no soldier*, or *every soldier*.

Crucially, although *he* cannot be co-valued with *no soldier*, it can depend for its interpretation on the latter. This is shown in (10):

(10)*No soldier* who has a gun hopes *he* will shoot.

That is, in (10) *he* can be semantically bound by *no* soldier. The semantic structure of (10) can be represented as in (11) where *he* is translated as a variable—x:

(11) No soldier who has a gun (λx.(x hopes [x will shoot]))

Under this construal the dependency of *he* on *no soldier* makes perfect sense. Here we have the relation of argument binding (A-binding), defined in terms of “logical binding,” as in (12):

(12) A-binding (Reinhart, 2006)
α is A-bound by β iff α is the sister of λ-predicate whose operator binds β

A crucial difference between coreference and A-binding is that the latter, but not the former is subject to a structural condition, namely c-command. Briefly, as indicated by the definition in (12), the A-binder must be the sister of a constituent containing the bindee, as in (13):

(13) A-binder […. bindee…]

The role of c-command is clearly illustrated by the contrasts in (14):

(14) a. The cop who found *the criminal* arrested *him*
b. *The criminal* found by the cop realized the latter would arrest *him*
c. ^*^The cop who found *every criminal* arrested *him*
d. *Every criminal* found by the cop realized the latter would arrest him
e. ^*^The cop who found *no criminal* arrested *him*
f. *No criminal* found by the cop realized the latter would arrest *him*

In (14a), *the criminal* does not c-command *him*, hence doesn't bind it, but since *the criminal* is referential and can have a discourse individual as its value, it can be co-valued with *him*, and there is no difference with (14b) where *the criminal* c-commands the pronominal; that is in both cases can *him* end up as covalued with *the criminal*. In (14c) *every criminal* does not c-command *him*, hence cannot bind it, hence the contrast with (14d). The same contrast is found between (14e) and (14f). Note, that one might argue that (14d) has a shallow counterpart that results by replacing *every criminal* by *all criminals*, and *him* by *them*, and possibly subjects might accept (14c) under such a construal (giving up on distributivity effects). But (14e,f) pose an insurmountable challenge to any such strategy; there is simply no alternative to a procedure in which *him* relates to the expression *no soldier*, and derives its interpretation from the instructions for interpretation this expression contains, since there is no discourse individual it could shallowly access instead^5^.

In summary, these contrasts show that two different modes of interpretation must be distinguished: (1) (*shallow*), directly assigning two (or more) expressions the same discourse entity from the interpretation domain (I_D_) as a value: *co-reference* as in (15a), and (2) (*deep*), interpreting one of the expressions first via another expression by grammatical—more specifically, semantic—means, as in (15b): *binding*^6^.

(15) 
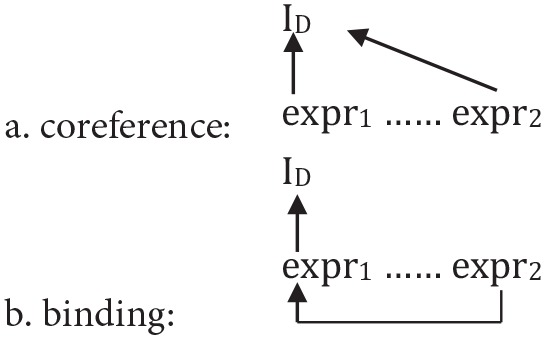

It should be clear from these considerations that the contrast between co-reference and binding proves that a certain type of *bottom-up* (deep) dependency, namely binding, is plainly impossible to represent *top-down* (shallowly), without recourse to grammatical computation. Thus, the *equivalence* assumption underlying the shallow approach does not hold—i.e., in the domain of anaphora there is for principled reasons no extra-grammatical alternative to binding. This brings us to the discussion of the *shallow advantage* assumption—or more specifically, the *shallow-by-top-down (shallow-TD) advantage*—which cannot be refuted as easily on theoretical grounds alone, but requires recourse to an accumulating body of experimental evidence.

## The shallow-TD advantage assumption: preparing the ground

While binding is subject to grammatical conditions, as we saw in (14), co-reference, by its very nature is not. Furthermore, binding is not only subject to the c-command requirement but elements to be bound can also be subject to a locality condition as illustrated in (16), with binding represented by co-indexing.

(16) a. Alice_i_ expected [the King to admire her_*i*_]
b. ^*^Alice expected [the Queen_*i*_ to admire her_*i*_]

The upshot is that—as can be easily observed in the vast majority of languages—a pronominal may not be too close to its binder. In (16a) *Alice* is sufficiently far away from *her* to serve as its antecedent, but in (16b) *the Queen* is too close, matches in features, but yet is not allowed to bind *her*. This is one of the main patterns captured by Condition B of the Canonical Binding Theory (CBT, Chomsky, 1981, 1986)^7^:

(17) Condition B
A pronominal must be free (=not bound) in its governing category (= roughly, the domain of its nearest subject).

Condition B is a grammatical principle. But, one may wonder, why cannot the prohibition expressed by condition B be bypassed by using coreference? That is, even if *the Queen* in (16b) cannot bind *her*, why cannot the language system simply resort to the strategy (or top-down heuristic) in (15a), and assign the same individual to *the Queen* and to *her*? If this would be possible, we would never see the effects of condition B with referential antecedents, contrary to fact. This led to the postulation of what one may call a “traffic rule,” reflecting an economy principle: *If a particular interpretation is ruled out by the grammar, this prohibition may not be bypassed* (see Reinhart, 1983; Grodzinsky and Reinhart, 1993; Reinhart, 2006; Reuland, 2011a, for discussion of this principle in various forms). In short, there are sentences where a binding and a coreference construal potentially compete, and when the binding dependency is rejected by the grammar, the coreference alternative is not considered. This is indicative of an economy ranking: *grammar* < *discourse*, reflected in Reinhart's Rule I and its successors^8^.

The notion of an economy ranking plays an even more crucial role in the Primitives of Binding (PoB) model developed in Reuland (2001, 2011a), where the conditions on binding are derived from more elementary properties of the grammatical system. In its simplest form this economy measure is based on the assumption that the language system as whole is “lazy” and prefers to minimize the number of cross-modular steps, as in (15b) with one cross-modular step less than (15a)—i.e., in (15a) more information needs to be transferred from the grammar system to the interpretational system than in (15b).

The dependencies discussed so far were established in the “translation procedure” from syntactic representations to the interpretation system. But dependencies can also be pre-encoded by morpho-syntactic means. Quite characteristically, morpho-syntactic dependencies are obligatory. Whereas *him* in (14c) cannot depend for its interpretation on *every criminal*, nothing prevents it from being interpreted as some individual in the associated discourse. This is different from what we see with anaphors, like English *himself*, Dutch *zich(zelf)*, etc. Such expressions *must* be bound (at least in the core cases, see Reinhart and Reuland, 1993). Moreover, they must be bound in a very local domain, as illustrated for English by the contrast in (18), here again represented with the index notation:

(18) a. ^*^Alice_*i*_ expected [the King to admire herself_*i*_]
b. Alice expected [the Queen_*i*_ to admire herself_*i*_]

In (18a) *Alice* is too far away from *herself* to serve as its antecedent, whereas *the King* is not a suitable antecedent due to a gender mismatch. As a result the sentence is ungrammatical. In (18b) *the Queen* is near enough, matches in features, and hence, binds *herself*. This is one of the main patterns captured by Condition A of the Canonical Binding Theory (CBT, Chomsky, 1981, 1986):

(19) An anaphor is bound in its governing category (= roughly, the domain of its nearest subject).

A characteristic property of the CBT is that it was based on a mix of syntactic and semantic properties. The notion of governing category is syntactic, the notion of binding itself is semantic, and the notion of an index—one of its key ingredients—was of a hybrid syntactic-semantic nature. This made it highly problematic as an ingredient of an explanatory theory (see Reinhart, 1983, for an initial discussion, and Reuland, 2011b, for a systematic overview of the problems with indices).

Minimalist approaches to grammatical structure (Chomsky, 1995, and subsequent work) introduced a strict separation between morpho-syntax and the interpretive system. Indices are not morpho-syntactic objects, hence, it was concluded, they have no place in syntax. Consequently, whatever there is syntactic in the binding conditions—such as locality—has to be derived with purely syntactic means. The means to do so in syntax are limited, just Movement and Agree (feature checking and valuation). This necessitated a thorough rethinking of binding and the binding conditions. A specific proposal to implement this was developed in Reuland (2001), and elaborated in Reuland (2011a). For reasons of space we will limit the discussion here to a few key issues, starting with condition A of the CBT.

In short, in Reuland (2011a) the locality property of *himself* is shown to follow from the semantic fact that *self* is an inherently reflexive relational noun. Given this property, *self* reflexivizes the predicate of which *himself* is an argument by—covert—head movement onto the verb (that is, it is interpreted as a reflexivizing operator). As we independently know, head-movement is strictly local (Travis, 1984). Hence, the locality of *himself* follows from the locality of head-movement. Thus, the relevant aspect of the representation of (18b) is as in (20):

(20) Alice expected [the Queen to SELF-admire her(self)]^9^
…. the Queen (λx. (x admires SELF (x))

The upshot is, then, twofold. First, the interpretation of *himself* /*herself* involves a purely syntactic movement operation. Second, it is not just a matter of *himself* /*herself* 'looking for an antecedent' and being valued by the latter, but the process crucially involves the reflexivization of the predicate.

Indeed, there is independent experimental evidence that the processing of SELF-anaphors involves the verb in addition to whatever properties the antecedent may contribute. For example, Manika (2014), and Manika et al. (2014) using an information-theoretic approach (see Kostić, 1991, 1995 and subsequent work) show that the interpretation of a referentially dependent lexical item like Dutch *zichzelf* is modulated by the complexity of the verb—as quantified by the inflectional entropy of its paradigm—indicating that the interpretation of *zichzelf* involves an operation on the verb itself^10^.

Also the binding of simplex anaphors like Dutch *zich* in (21) is encoded in the syntax (where SE stands for *simplex element anaphor*):

(21) *De klimmer* voelde [*zich* wegglijden]
The climber felt [SE slip away]

Here the encoding is brought about by the operation Agree: *zich* is deficient for gender and number, and is valued by Agree copying these features from the antecedent onto *zich*. The fact that (22) is ill-formed, again follows from economy (this contributes to deriving the canonical Condition B).

(22) ^*^De klimmer voelde [*hem* wegglijden]
The climber felt [him slip away]

To account for the fact that (22) is ruled out we apply the same logic as in the case of Rule I. The anaphoric dependency between *de klimmer* and *zich* in (21) can be encoded in syntax by Agree, but now consider the case where *hem* is selected, as in (22). Since *hem* is fully specified, it has no empty cells. Consequently, valuing it in the syntax by Agree is not an option. Hence, *zich* wins. But, crucially, this can only work if syntax cannot be bypassed by a derivation in which *hem* is directly interpreted as a bound variable by applying (12). So, syntax has to be considered before semantic binding can apply, and if syntax rejects the derivation, this is final. Since a syntactic operation such as Agree operates locally, we see this competition only when the dependent element is within the Agree domain of the element it is to depend on and not when it is further away^11^. Consequently, we arrive at the economy ranking in (23).

(23) syntax < semantics < discourse

We have by now prepared the ground for a discussion of the second assumption of good-enough interpretations: Are, deep—grammatical—operations indeed more costly for the processor than shallow-TD operations (i.e., in contrast to the economy ranking as depicted above)?

## The shallow-TD advantage assumption: the issue of economy

Interestingly, the issue of economy has received quite a bit of attention in the experimental literature, though not from the perspective sketched in the current contribution.

### The economy of syntax

As is well-known, research on language acquisition shows an asymmetry between the performance of young children on condition A as compared to condition B. For instance, Chien and Wexler (1991) explored the question of whether children know Principles A and B from the outset or not. Their experiments show that children correctly require *local* antecedents for reflexives (Principle A) early on, whereas they are significantly delayed in disallowing *local* antecedents for pronouns (Principle B). As argued in Grodzinsky and Reinhart (1993) the computations involving the correct application of condition B are more costly than those involved in condition A. From the present perspective this indicates that the syntactic mode of encoding is indeed the least costly^12^.

Although it is generally assumed in the psycholinguistic literature that condition A is a syntactic condition, it may be good to point out that in the PoB system condition A, as it is reinterpreted, is indeed a purely syntactic operation (of course with semantic consequences). Hence, it is an ideal testing ground for the shallow vs. deep processing issue.

There are a number of experiments reported in the literature that test the status of condition A. Crucial for the present discussion, their results indicate that condition A applies early in the time course of processing and is, in addition, very robust. To illustrate this, in a well-known study Sturt (2003) carried out two eye-tracking experiments measuring (mis)match effects in sentences such as (24):

(24) *Jonathan/Jennifer* was pretty worried at the City Hospital. *He/She* remembered that *the surgeon* had pricked himself/^*^herself with a used syringe needle. There should be an investigation soon.

In all the conditions only one character was structurally available (i.e., *the surgeon*, a profession with a stereotypically male gender). The distracting character (i.e., *Jonathan* or *Jennifer*) was highly prominent in the preceding discourse, yet not accessible as an antecedent for the reflexive. The results showed that if the reflexive and structurally available antecedent differed in gender, this immediately slowed down the reading process. Moreover, at this point during processing the distracting character (i.e., *Jonathan/Jennifer*) did not influence the resolution process. This suggests that the language system first attempts to link the reflexive to an antecedent that is structurally available—which will immediately fail when there is a gender-mismatch. In a follow-up experiment Sturt modulated the relative position of the distractor in the sentence, but the same conclusion was supported.

These finding are on a par with several other studies adopting a wide range of methodologies. For example, in an ERP experiment where the participants processed sentences such as (25), Xiang et al. (2009) investigate “intrusion effects” of potential, but non-commanding antecedents that appear—intrude—on the path between the SELF-anaphor and its antecedent.

(25) a. Congruent
The tough soldier that Fred treated in the military hospital introduced himself to all the nurses.
b. Intrusive
The tough soldier that Katie treated in the military hospital introduced herself to all the nurses.
c. Incongruent
The tough soldier that Fred treated in the military hospital introduced herself to all the nurses.

Furthermore, they compared these conditions to paired conditions in a second ERP-experiment in which intruders were present on the path between Negative Polarity Items and their licensers. Although they did find intrusion effects in the latter case, no significant intrusion effects were obtained in the case of the SELF-anaphor conditions as presented above (i.e., the ERP-waveforms revealed no difference between condition b and c). They concluded that during reflexive binding, syntactic constraints appeared to prevent intrusive antecedents from influencing the initial stages of anaphor resolution. In our view this points toward an early and robust application of the syntactic process establishing the dependency.

As a final example, Cunnings and Felser (2013) investigated the processing of SELF-anaphors in English, using the eye-tracking methodology. In their experiments they compared the performance of low working memory span with high working memory span readers. Here we will focus on one experiment—their Experiment 2—in which they measured the effect of a linearly intervening—but inaccessible antecedent (due to lack of c-command) using sentences as in (26):

(26) *James/Helen* has worked at the army hospital for years. *The soldier* that *he/she* treated on the ward wounded himself/^*^herself while on duty in the Far East. Life must be difficult when you are in the army.

If Principle A would reflect a processing-based constraint this would lead to a different prediction than if it were a purely syntactic constraint. In the former case, particularly lower span readers may initially attempt to keep referential dependencies as short as possible. If so, main effects of the inaccessible antecedent should initially be observed. Higher span readers, on the other hand, would be less likely to find the creation of longer anaphoric dependencies difficult. It was found that for both lower and higher span readers the online application of Principle A could not be reduced to a (shallow-TD) memory-friendly “least effort” strategy of keeping anaphoric dependencies as short as possible^13^. All in all, the joint results of the two experiments they reported support, as they put it, a growing body of evidence showing that binding Principle A applies early during sentence processing to help guide reflexive anaphor resolution (e.g., Nicol and Swinney, 1989; Felser et al., 2009; Felser and Cunnings, 2012; Xiang et al., 2009; but see Badecker and Straub, 2002, for some conflicting evidence; see Dillon, 2014, for an excellent overview of all the relevant results).

Hence, a preferential position of syntactic encoding with respect to other strategies of anaphora resolution is warranted, which is in line with the PoB model (but not predicted by other approaches to binding). Or to put it slightly differently, a deep syntactic operation like binding of a SELF-anaphor is less costly for the processor than shallower operations, in contrast to what the “shallowness” approach predicts. In fact, this already is sufficient to establish our main point. There is *no clear support for a shallowness advantage*. Rather the opposite is the case: for the human processor *deep syntactic computations are preferred over shallow-TD interpretation processes*.

However, it will nevertheless be important to also assess the other members of the economy hierarchy as formulated in the PoB model: binding and coreference. This is what we will do next.

### The economy of binding and coreference

A well-known instantiation of the (economy) contrast between binding and coreference, introduced in (23) above, shows up in the interpretation of sentences with VP ellipsis, as in (27):

(27) John fed his cat and Peter did too

Before we elaborate on this contrast in terms of economy, however, some facts and assumptions on VP-ellipsis should be discussed. First of all, it is clear that the second conjunct is about Peter feeding a cat, rather than about him combing a dog. This, uncontroversially, is a fact any theory of language will have to capture. A common idea is that for interpretation to obtain, the content of the VP in the second conjunct must somehow be recovered from the preceding context. As a first go one may assume a copying operation, as in (28).

(28) John fed his cat and Peter did < feed his cat> too.

As one can see, this gives rise to a puzzle, since the elided (i.e., covert) pronominal *his* in the second conjunct is ambiguous. More specifically, the interpretation of the full sentence can be either that John fed John's cat and *Peter fed Peter's* cat, as in (29a), or that John fed John's cat and that *Peter also fed John's cat*, as in (29b):

(29) a. John (λx. (x fed x's cat)) & Peter (λx. (x fed x's cat))
b. John (λx. (x fed a's cat) & a=J) & Peter (λx. (x fed a's cat) & a = J)

In (29a) *his* is interpreted as a variable, x, A-bound by Peter. This is what is generally referred to as the *bound variable* (BV), or “sloppy”^14^ interpretation. In (29b), however, *hi*s is interpreted as a constant, here represented as *a*, which can receive the value of any individual in the discourse including John. That is, the occurrences of *his* in both conjuncts are *coreferential* (COR), yielding a “strict” interpretation.

Interestingly, the human processor is sensitive to this difference, and more importantly, it is a consistent finding in offline studies that in the interpretation of ambiguous VP-ellipses, BV-based interpretations are preferred over COR interpretations (see Frazier and Clifton, 2000, for an overview). This “preference” is reflected in the fact that typical subjects show longer reading times on COR in self-paced reading experiments (reported in Frazier and Clifton). In another experiment on the interpretation of VP ellipses that involved subjects with agrammatism, these subjects performed 80% correct on BV interpretations, but at chance on COR interpretations (Vasic et al., 2006). Curiously, then, what might seem to be the less sophisticated—more shallow—procedure, is the one that comes out as more costly in this case as well.

On the basis of such findings Frazier and Clifton (elaborating Reinhart, 1983; Avrutin, 1994, 1999) propose the following thesis as a hypothesis worth exploring:

(30) LF only/first hypothesis:
Bound-variable interpretations are preferred because the perceiver need only consult the LF representation (not the discourse representation) in order to identify the bound-variable analysis of the sentence.

In order to do so they carry out a number of exploratory experiments and conclude that the hypothesis, though compatible with some of their results, is too problematic to be maintained.

However, as discussed by Frazier and Clifton (see also Koornneef, 2008; Koornneef et al., 2011) their results should be interpreted with some care, due to limitations of the experimental design and the statistical evaluation. In order to obtain more dependable results, subsequently, a number of full-size experiments using a more sensitive methodology were carried out, reported in Koornneef (2008, 2010), and Koornneef et al. (2006, 2011). Since the case is illustrative of the need to take theoretical advances into account we will briefly discuss Frazier and Clifton's interpretation of their findings before turning to the experiments of Koornneef and his colleagues.

One of the problems Frazier and Clifton note is of a *theoretical* nature. As they observed, a BV-preference also obtains across sentence boundaries, as in (31) (Experiment 1b). According to Frazier and Clifton this is incompatible with the nature of LF operations. That is, one would expect a grammatical operation like VP-copying to be limited to the domain of a sentence.

(31) Sarah left her boyfriend in May. Tina did [leave her boyfriend] too.

The other problem is *empirical* in nature. The choice between variable binding and coreference also shows up in the interpretation of *only*-sentences, illustrated in (32). Here it concerns the interpretation of the pronominal *he* in the complement clause of *think*. And again the pronoun shows an ambiguity. However, contrary to VP-ellipsis, Frazier and Clifton find a preference for a COR interpretation instead of the BV interpretation.

(32) Only Alfred thinks (that) he is a good cook.
a. Only Alfred thinks that Alfred is a good cook. (COR)
b. The only person who thinks of himself as a good cook is Alfred.
(BV).

On the basis of these findings, Frazier and Clifton conclude that the LF-only hypothesis (and equivalents) cannot be maintained. This, however, leaves a puzzle. Why would the case of VP-ellipsis be different from the *only*-case and what conclusions should we draw about the language processing system? Let's first address the theoretical issue Frazier and Clifton raise.

#### Theoretical issue: what mechanism underlies ellipsis?

The mechanism originally assumed in the literature on VP-ellipsis since Hankamer and Sag (1976) involved a copying operation (see Elbourne, 2008, for an overview and references). If so we would have to assume that the empty VP in the second sentence in (33a)—indicated by Δ—would be filled by a syntactic operation applying across sentences.

(33) a. Sarah left her boyfriend in May. Tina did Δ too.
b. Sarah (λx. (x left x's boyfriend)). Tina (λx. (x left x's boyfriend)) too.

This, Frazier and Clifton feel, violates the generally accepted idea that grammatical operations are limited to the sentential domain. Therefore, Δ cannot be interpreted by a grammatical copying operation. The question is, then, what kind of mechanism, is involved.

In recent years, however, independent evidence has been found that the theory of ellipsis should allow for greater flexibility (Merchant, 2001, 2008; Elbourne, 2008). This is illustrated by cases like (34) (Elbourne, 2008):

(34) Saskia, being a competitive type, has managed to acquire all the skills that Maaike and Brigitte possess. Maaike dances. Brigitte sings. Saskia does Δ too.

Here, Δ can be interpreted as the combined property of singing and dancing. In order to account for these and a variety of other cases, Elbourne proposes that ellipsis sites have internal—unpronounced—syntactic structure and are to be analyzed as silent “definite descriptions.” In line with this, (33a) would be represented as (35), where the label TheP indicates that the complement of *did* is such a silent definite description (perhaps superfluously, we also indicate the silence by strike-through).

(35) Sarah left her boyfriend in May. Tina did [_TheP_ leave her boyfriend] too.

Then, to interpret the VP-ellipsis, the parser must somehow access the context (in this case “Sarah left her boyfriend in May”) to retrieve the values for the constituent parts of TheP. Elbourne provides an elegant, yet fairly extensive and technical implementation whose details are beyond the scope of our present contribution. Relevant here is that, as he shows, the interpretation of the ellipsis site does not depend on a sentence-grammar “copy-and-paste operation,” but rather reflects how a pronominal picks up its reference. That is, the elided VPs are treated as null pronouns, and under anybody's account, pronouns are able to pick up values from the preceding context. Hence, the relevant difference with the LF copying account is that under Elbourne's approach there is no theoretical reason to expect the context for the interpretation of VP-ellipsis to be limited to the same sentence.

What does the above mean for the explanation of a BV preference in VP-ellipsis like (34) in which the interpretation of the section “Tina did too” depends on retrieving information from a previous sentence? In fact, given that Elbourne's account obviates the same-sentence constraint, the same mechanisms are at work as in (28) where the elided site and the context clause *are* part of the same sentence. To illustrate this, in (35) the parser retrieves either “leave x's boyfriend” as value for the TheP (i.e., the preferred BV interpretation), or alternatively, it picks up “leave Sarah's boyfriend” as a COR alternative. More specifically, just like in the classic examples of VP-ellipsis—in which the ellipsis and context clause are part of the same sentence—any preference for a dependency type in the first sentence will be inherited by the second sentence in (35). No additional stipulations are necessary and in fact the theoretical problem as described by Frazier and Clifton does not arise—which illustrates yet again the fact that it is important to keep reassessing the interpretation of experimental results in view of theoretical advances^15^.

#### Empirical issue: interpretational preferences in only-sentences^17^

In addition to a theoretical problem for the BV-preference in VP-ellipsis, Frazier and Clifton also report an empirical problem for so-called *only*-sentences. In order to understand what is at stake in *only*-sentences, consider again the pattern in (32), repeated here with additional material:

(36) Only Alfred thinks (that) he is a good cook.
a. Only Alfred thinks that Alfred is a good cook (COR)
Only Alfred (x thinks Alfred is a good cook)
b. The only person who thinks of himself as a good cook is Alfred. (BV)
Only Alfred (x thinks that x is a good cook)

Frazier and Clifton conducted a questionnaire study, which shows a strong preference for the (36a) interpretation among the respondents. However, there is a caveat about such off-line studies. They reflect an end-result, but don't give insight in the process itself. As it is, if we wish to interpret their results two questions come up. First, is it just a matter of BV vs. COR, or do other factors play a role? Second, what kind of information does the language processor have to draw together, to obtain either a BV or a COR interpretation in sentences with *only?*

For a proper understanding of these issues at least the following crucial fact should be taken into account: Across both interpretations the fact that Alfred is happy about his own cooking remains constant. Yet, a full interpretation requires the representation of some sort of “hidden” reference set consisting of everybody but Alfred, or in other words the *contrast set* (e.g., Rooth, 1985). The contrast set, implicitly introduced through the use of the term *only*, behaves differently in a BV reading than in a COR reading: whereas in the BV reading each individual member of the set is not that happy about his own cooking, the contrast set in the COR reading consists of members who think that Alfred's cooking is not very good. Given this, a possible additional factor in a BV or COR preference is how well the hidden contrast set fits the context overall.

Thus, a factor to take into account is that, possibly, the hidden set of the COR reading in the sentences tested by Frazier and Clifton just fits the context better. In fact, Frazier and Clifton presented their sentences without an explicit context. But, in order to interpret *only*-sentences, participants will have to set up a context. Thus, the question is what context they construe.

Crain and Steedman (1985) propose a *Principle of Referential Success*, reflecting that people choose the reading with the fewest “open ends.” In view of this, it may well be the case that a strict interpretation is chosen more often in “*only* Alfred thinks he is a good cook” because it is more likely that the sentence is talking about Alfred's cooking, which is explicitly mentioned, than about the cooking of the “entire world.” Hence, the lack of context could very well bias participants to a COR interpretation regardless of whether the language processor initially prefers a BV reading or not. It is therefore crucial to properly investigate the role of context, and, where necessary, control for its effects.

In summary, Frazier and Clifton (2000) reported both a theoretical problem and an empirical problem for the LF-only hypothesis—which incorporates the BV preference. We have shown that the theoretical problem with VP-ellipsis is in fact not problematic according to the most recent insights of linguistic theories. The second problem (a COR preference in only-sentences), we argued, required further testing. More specifically, as we will discuss in the next section, it generated the following hypotheses in (37) and a series of experiments testing them (e.g., Koornneef et al., 2006, 2011; Koornneef, 2008, 2010; Cunnings et al., 2014).

(37) Hypotheses
- The language processor initially prefers a BV interpretation.
- Context may then lead to a COR interpretation.
- This (mental) backtracking should be visible in the time course of the process.

#### Tracking the time course of anaphora resolution

The hypotheses presented in (37), and the issues raised by Frazier and Clifton regarding sentences containing the only-operator, were addressed by Koornneef et al. (2011) in a questionnaire (to assess the final interpretation of the participants) and an eye-tracking experiment (to track the mental processes preceding this final interpretation). In their study Dutch university students read a series of short texts in 4 versions about 2 story characters of the same gender (e.g., *Lisa* and *Anouk*, see ex. 38).

(38) Example of BV-biased/only-sentence condition
(S1)Lisa en Anouk zijn dol op de muziekzender MTV. (S2) Zij konden hun geluk niet op toen zij mee mochten doen aan het programma “Pimp My Room,” waarin hun kamers werden opgeknapt. *(S3) Alleen Lisa vindt dat haar gepimpte kamer klasse heeft.* (S4) Smaken verschillen nu eenmaal.
“(S1). Lisa and Anouk love the music channel MTV. (S2) They were very happy when they were selected for the show ‘Pimp My Room,’ in which their rooms were redecorated. *(S3) Only Lisa thinks that her pimped room has a touch of class.* (S4) Oh well, each to his own taste.”

Each story contained a critical third sentence (S3) that was ambiguous between a sloppy (BV) and strict (COR) interpretation. Moreover, two factors were manipulated in the stimuli. First, the critical sentence was an ambiguous only-sentence (e.g., “Only Lisa thinks that her pimped room has a touch of class.”) or, alternatively, an ambiguous VP-ellipsis sentence (e.g., “Lisa thinks that her pimped room has a touch of class, but Anouk does not”). Second, by providing background information in the second sentence about both story characters (“Lisa and Anouk were very happy…”) or, alternatively, about only one story character (“Lisa was very happy…”), the context either favored a BV interpretation or a COR interpretation of the ambiguous critical sentence, respectively.

The results of the questionnaire experiment, in which the participants presented their final interpretation of the ambiguous sentence (in addition to providing ratings of story-plausibility and -difficulty) showed that, while using a relatively, simple manipulation and exactly the same critical sentence, readers were more easily biased toward a BV interpretation than toward a COR interpretation. Moreover, contrary to the findings of Frazier and Clifton the context manipulation in the second sentence affected the interpretation of the only-sentences and ellipsis-sentences in the exact same way. Hence, these finding are consistent with the idea that the interpretation of the referential ambiguity in only-sentences and VP-ellipses is driven by the same constraints, which preferable single out a BV interpretation.

The eye-tracking data of the reading experiment of Koornneef et al. (2011) confirmed and extended these results. First of all, the stories in which the interpretation of the ambiguous sentence was biased toward a BV interpretation elicited shorter first pass reading times in the critical VP-ellipsis sections than the stories biased toward a COR interpretation^18^. Furthermore, the reading times for the second sentence (i.e., the sentence that contained the biasing information) also revealed a clear contrast between the COR- and BV-biased stories. In this case the second-pass durations—indicative of re-analysis and repair—were much longer for the COR-biased stories. Interestingly, this was observed for ellipsis- and only-sentences alike, which again suggests that the preference for BV interpretations is not restricted to ellipses, but a general property of the parser.

In all, the results of the offline questionnaire and in particular the online eye-tracking experiment were consistent with the hypotheses as formulated in (37). That is, the readers initially preferred a BV reading, since BV reflected the cheaper option in the processing hierarchy. However, when the larger context forced a COR reading instead, readers reanalyzed the story to change their initial BV reading into the more suitable COR reading. This (mental) backtracking surfaced in the eye-tracking data as longer first-pass reading times near the elided section of the ellipsis sentences and longer second-pass reading times at the biasing second sentence.

In a similar eye-tracking study examining the interplay between BV and COR, Koornneef et al. (2006) showed that the preference of the parser for BV dependencies generalizes beyond ambiguous ellipsis- and only-sentences. They observed that in sentences like (39) containing a quantified antecedent “iedere arbeider” (every worker) in a c-commanding position and a proper name “Paul”in a non-commanding position, readers more easily connected the ambiguous pronoun to the former than to the latter—even when the context preceding the critical sentence clearly mandated the COR reading in which “hij” (he) equaled “Paul.”

(39) *Iedere arbeider* die zag dat *Paul* bijna geen energie meer had, vond het heel erg fijn dat *hij* wat eerder naar huis mocht vanmiddag.
“Every worker who noticed that Paul was running out of energy, thought it was very nice that he could go home early this afternoon.”

In a more recent eye-tracking study, however, Cunnings et al. (2014) addressed some weaknesses in the stimuli of Koornneef et al. (2006) and failed to replicate the preference for quantified c-commanding antecedents over non-c-commanding proper names. More specifically, in the most relevant experiment of their study (i.e., Experiment 1) Cunnings et al. embedded sentences like (40) in a short discourse and manipulated the gender of the critical pronoun and the preceding proper name^19^.

(40) *Every soldier* who knew that *James/Helen* was watching was convinced that *he/she* should wave as the parade passed.

At the critical pronoun and the region immediately following the pronoun they observed longer re-reading and total reading times when the proper name antecedent mismatched in gender with the pronoun. These results, according to Cunnings et al., indicated that readers preferred to connect the pronoun to the linearly closer, yet non-c-commanding antecedent. This would be inconsistent with the PoB framework, since “it fails to support the hypothesis that variable binding relations are computed before coreference assignment.”

Although we agree with Cunnings et al. that these results do not provide strong evidence in favor of the PoB approach we disagree with the claim that the results are inconsistent with the approach, for the following reasons. First, in the experiment of Cunnings et al. the individuals [James/Helen in (40)] were not introduced previously—note that this was controlled for in the Koornneef et al. study (2006; see for a detailed discussion Koornneef, 2008). Therefore it is not unlikely that the readers were trying to get further information after the topic shift in the story, and thus tempted to consider a subsequent pronominal as a source of such information. This would be consistent with the fact that the reported differences show up in so-called “later” eye-tracking measures only. Which brings us to a second and arguably more important issue. That is, since the reading time differences become visible in later eye-tracking measures only, the non-c-commanding proper name does not seem to impact the interpretive costs of the pronoun immediately. Hence, instead of ruling out an early preference for BV dependencies over COR dependencies, the findings of Cunnings et al. indicate that COR distractors can influence the interpretive system during later stages of processing—i.e., not unlike the defeasible filter model concerning Principle A (e.g., Sturt, 2003). Crucially, this would be compatible with the PoB approach in which the choice between variable binding and coreference for an ambiguous pronoun is intrinsically free (e.g., Koornneef, 2008).

In all, we do not fully agree with the conclusions as presented by Cunnings et al. (2014), and hence, we maintain our position that there is sufficient evidence for a BV preference—and no convincing evidence against it. Hence, with respect to the good-enough approach (e.g., Ferreira, 2003; Karimi and Ferreira, 2015), the focus of our current contribution, we state that the empirical studies examining bound vs. coreferential dependencies confirm and extend our previous conclusion, where we reported that grammatical operations (such as binding of a SELF-anaphor) are less burdensome for the processor than shallower operations. Again in contrast to what the good-enough approach predicts, the experiments discussed above show that the same holds for binding of a pronominal; the deep variable binding algorithm is less costly than—and preferred over—the shallow top-down driven operation of coreference^20^.

Before we present our final assessment of the good-enough approach in the domain of anaphoric dependencies, however, we should address some interesting suggestions of Cunnings et al. (2014) as to how their results can be related to more general architectural issues. First, they observe that a recurrent issue, highly relevant for the bound variable vs. coreferential (or grammatical vs. extra-grammatical) distinction, is the role of structure-based vs. unconstrained cue-based memory retrieval mechanisms (see e.g., Dillon, 2014; Jäger et al., 2015a, for recent overviews of this issue). Second (and somewhat related), they suggest that their results are more easily explained with a uni-modular approach as in Heim (2007), than with the multi-modular architecture assumed in the PoB model. These two architectural issues will be addressed in more detail below.

### Structure-based vs. unconstrained cue-based retrieval

The PoB economy ranking in relation to shallow vs. deep processing is by no means the only issue that arises in the field of anaphor processing. For example, by now an important recurrent issue—although to some extent orthogonal to the economy issue—is what kind of retrieval mechanism promotes anaphor resolution. More specifically, based on a growing body of literature, Cunnings et al. (2014) distinguish two theoretically plausible ways in which the antecedent of a linguistic element can be retrieved from (working) memory. As a first possibility, a serial search mechanism is proposed in which the text representation is searched in a step-by-step manner until the proper antecedent for an anaphor has been located. A qualitatively different search (or retrieval) mechanism is based on the idea of a content-addressable memory (CAM) architecture (Lewis et al., 2006). In the latter type of memory systems, previously stored information can be accessed directly by the use of certain features as retrieval cues.

Cunnings et al. (2014; see also Jäger et al., 2015a,b) make the interesting conjecture that a specific instantiation of a serial search mechanism could be a structure-based retrieval mechanism in which syntactic tree-configurational information (e.g., c-command) guides the retrieval process. That is, in these type of systems “the priority in which antecedents are retrieved is dependent upon their relative position in the search path” (pp. 42) which would be compatible with an architecture assuming a BV preference. In contrast, CAM-like, unconstrained cue-based retrieval assumes that all available cues (e.g., gender, number, person, animacy, etc.) are used immediately (and in parallel) to retrieve an anaphor's antecedent. This system allows for more flexibility as structural constrains do not have a privileged status and, hence, COR interpretations of (reflexive) pronominals are also considered immediately—i.e., not subsequent to BV interpretations.

Cunnings et al. (2014) claim that the results of their eye-tracking experiments favor the latter cue-based approach, as recency (or linear proximity) of the antecedent seemed to guide the resolution process of a pronoun, rather than the structural notion of c-command. Indirectly, then, one could state that there is no solid experimental evidence to maintain a distinction between variable binding and coreference (cf. our discussion on uni-modular vs. multi-modular architectures below). Moreover, it would imply that the same cue-based memory mechanisms underlying the construction of a range of other (syntactic) dependencies—such as filler-gap dependencies (McElree et al., 2003), subject-verb dependencies (Van Dyke and Lewis, 2003; Van Dyke and McElree, 2006; Van Dyke, 2011, 2007; Wagers et al., 2009; Dillon et al., 2013), the licensing of negative-polarity items (Vasishth et al., 2008) and verb-phrase ellipsis (Martin and McElree, 2008)—are responsible for determining the proper antecedent for (reflexive) pronominals.

Whether cue-based memory retrieval, however, is indeed the most valid way to describe anaphoric processing is hotly debated still. For example, Dillon (2014) shows in a very systematic overview that reflexives are relatively immune to so-called retrieval interference, a property that would set them apart from superficially similar syntactic dependencies like subject–verb agreement. This conclusion in turn, is disputed by Jäger et al. (2015a) who conducted reading time experiments on German and Swedish reflexives, and *did* observe occurrences of retrieval interference as predicted by the cue-based approach—and as they claim, not by the structure-based approach.

Hence, at this point in time we are simply not in the position to single out a unique framework as the correct approach. In fact, in the case of anaphora it might well be true that both types of memory retrieval systems are somehow involved. For one thing, although binding dependencies are often discussed in terms of c-command, this certainly does not entail that the formation of logical form representations should be considered to be blind to cues such gender and number. Hence a possible, and in fact very plausible, outcome is that the antecedents for (bound) pronouns are determined by means of a system that combines structure- and cue-based search algorithms, with their respective roles depending on timing. For instance, one might expect intrusion effects at a stage before the final structure is established. In all, the precise nature of the interplay between c-command vs. morpho-syntactic cues is an important issue that must be left for future research (but note that coding a tree-configurational relation as a cue for a CAM-like system is not as straightforward as coding gender and number; see Jäger et al., 2015a, footnote 4).

Albeit in a different way, this latter question also surfaces in the second architectural issue raised by Cunnings et al. (2014). That is, incorporating c-command as a “normal” cue in a CAM retrieval system, or alternatively, setting it apart as a qualitatively different cue, can ultimately be interpreted as a debate on uni-modular vs. multi-modular approaches to anaphor resolution.

### Uni-modular vs. multi-modular architectures

A very fundamental issue raised by Cunnings et al. (2014), concerns the (uni)-modular architecture of the anaphoric system. That is, in contrast to the PoB framework (in which at least three different modules/algorithms are assumed to underlie anaphora interpretation) they follow Heim (2007) who, they claim, puts forward a uni-modular approach. However, we feel that their interpretation of Heim's proposal on uni-modularity is less straightforward than they assume.

First, Heim's discussion is limited to condition B, and the status of Reinhart's Rule I. It does not address condition A, which uncontroversially is syntactic. So, even if Heim's endeavor works for condition B, binding theory as a whole would still minimally be “bi-modular.”

Second, Heim does not include the interpretation of proper names and other referential expressions in her discussion. But, even in her system, one must assume that these are directly interpreted as some individual in the discourse—but of course, relative to context. This interpretation strategy, however, must also be available for certain uses of pronouns. Just like we can start a story with *Helen was watching the parade with a feeling of disgust. Suddenly ….*where we are introducing a discourse individual and slowly building a character while reading on, we can start a story with *She was watching the parade with disgust. Suddenly…* and again we will be introducing a discourse individual and slowly building a character. It seems to us that there is no independent ground to treat the reference assignment differently in these cases. If so, not all cases of pronominal interpretation will fall under the binding strategy Heim proposes. Hence, whatever the division of labor in other cases, no truly uni-modular model for this domain will result in the end.

Heim doesn't discuss this issue. But if one looks carefully, one sees that what she achieves is tantamount to building Reinhart's Rule I into the binding conditions. Given that she set out to retain the core of Reinhart's insight, it is not surprising, then, that it surfaces in the details of the formulation of condition B. In fact what her system does is generalize over the “worst case scenario.” The difference between binding and co-valuation shows up in the explicit role of context in the latter, but not in the former. This is interesting by itself, since from a processing perspective, this would make it quite unexpected for co-valuation to require fewer resources than binding. But it also shows that the core of the contrast between binding and co-valuation is in fact retained in her system.

Note furthermore that Heim's unification program is based on the idea that condition B is essentially semantic. However, as shown in Volkova and Reuland (2014), this idea cannot be maintained in view of languages with locally bound pronominals. Such cross-linguistic variation shows that there must be a syntactic component in condition B (see Reuland, 2011a, and Volkova and Reuland, 2014, for further evidence that condition B is in fact not a unified phenomenon). Pronoun resolution in such languages [as for instance Frisian, or (Tegi) Khanty] has not yet been studied experimentally to our knowledge. Such experiments could shed further light on the way interpretive dependencies are processed, and more specifically, on the contrasting economy rankings and its relation to shallow and deep processing as proposed in the good-enough and PoB frameworks.

This brings us back to the issue we started out with, and in fact to a conclusion^21^

## Conclusion

As part of our more general goal of reassessing the interpretation of experimental results in view of the ongoing advances made in theoretical linguistics and psycholinguistics, the main focus of the current contribution was to evaluate the core assumptions of the good-enough framework as proposed by Ferreira and colleagues (e.g., Ferreira, 2003; Ferreira and Patson, 2007; Karimi and Ferreira, 2015). We structured our discussion around a recent elaboration of the good-enough approach (Karimi and Ferreira, 2015) in which an explicit distinction is being made between “deep” bottom-up syntactic algorithms and “shallow” top-down semantic/discourse operations. Crucially, given the presumed complexity of syntactic algorithms, the latter type of (extra-grammatical) heuristics should be preferred, thereby inducing good-enough representations of an utterance or text.

As it turned out, one of the key-notions in the discussion had to be reassessed. That is, we proposed that one must make a distinction between *shallow-TD* processing as a top-down process, and *shallow-R* processing as involving a reduced input (see e.g., Stewart et al., 2007). Taking this into account, the conclusion in terms of the *shallow equivalence* and the *shallow advantage* assumptions (cf. Principle 1 and 2 in Karimi and Ferreira, 2015) as formulated at the outset of this contribution are straightforward and simple. First, in the domain of anaphoric dependencies the equivalence assumption does not hold. There are binding dependencies whose interpretation cannot even be approximated by shallow-TD procedures. Second, and perhaps for current purposes more importantly, we reviewed a variety of experiments bearing on a purported shallow-TD advantage. None of the experiments provided support for such an advantage. Rather the opposite is the case: in the domain of anaphoric dependencies deep algorithmic computations are preferred over shallow-TD interpretational processes. Such a preference not only shows up in the comparison between syntax and what one may broadly call the interpretive system, but also *within* the latter system, i.e., between deep, structure-based (variable binding), and shallower context-based (coreference) interpretive procedures.

There is one important proviso: as becomes clear from the discussion (e.g., regarding Heim, 2007) context-based interpretive procedures may in fact require more computation than meets the eye. Hence, properly considered, they may not be as shallow as they prima facie appear to be. Perhaps, then, they are more costly because they, at least in some cases, require more sophisticated computations. But if this is so, this casts doubt on the very idea that there are truly shallow procedures. Such shallow procedures may well be no more than illusory effects that arise if some material is not admitted into the buffer. Therefore, we submit the bold claim that, until proponents of the existence of shallow procedures offer precise and falsifiable descriptions, Occam's razor requires us to treat them as just that: illusions.

## Author contributions

All authors listed, have made substantial, direct, and intellectual contribution to the work, and approved it for publication.

## Funding

This work was supported by an NWO (Netherlands Organization for Scientific Research) Veni grant [grant number 275-89-012] awarded to AK.

### Conflict of interest statement

The authors declare that the research was conducted in the absence of any commercial or financial relationships that could be construed as a potential conflict of interest. The reviewer, CP, and handling Editor declared their shared affiliation, and the handling Editor states that the process nevertheless met the standards of a fair and objective review.

## Footnotes

^1^See Dąbrowska (2012) for the claim that such differences in interpretation reflect differences in acquisition, but Reuland (2012) for a demonstration that this claim is unwarranted.

^2^Or, hearers may adopt a simple Noun-Verb-Noun heuristic based on frequency patterns (Townsend and Bever, 2001). But this again raises non-trivial questions. That is, even if one assumes that this heuristic could be obtained on the basis of frequency—i.e., note that most canonical sentences only match this pattern if much material is ignored—it raises fundamental questions like, how is it stored, and how is it retrieved, and crucially how does it contribute to interpretation? In fact, it can only do so if the pattern is interpreted as “Subject Verb Object.” But these are not surface encoded notions, but in fact already presuppose non-trivial analysis.

^3^Note that this use of the term semantics is different from ours. Whereas semantics in the model we will present refers to logical form (LF) representations, the label semantics in Kuperberg (2007) and Karimi and Ferreira (2015) in part reflects lexical semantics, and in part would be classified as discourse representations in our model.

^4^Note that even the simplicity of a principle like *Agent first* is in fact not obvious. Properly considered it hides a considerable number of hidden assumptions. Consider, therefore, semantic role assignment in more detail: Task: Assign a semantic role to DP_1_

(i) DP_1_ …. [_VP_ V ….]

Options:

1. Apply compositional interpretation procedure to V, VP, and DP_1_
2. Go to extra-grammatical set of heuristics, find *Agent first* and assign Agent to DP_1_

The leading idea in heuristic based approaches is that option 2 is less costly than option 1. But the simplicity of Agent first is misleading. Consider the fuller structure in (ii):

(ii) DP_1_ was [_VP_ V-ed by DP_2_]

In order to get the relevant (wrong) interpretation the processor has to:

1. Leave grammar
2. Access list of heuristic principles
3. Find Agent first
4. Apply Agent first
5. Disregard functional material (was, -ed, by)
6. Apply the predication rule between DP_1_ and VP

But this raises issues such as:

- What do these heuristic principles arise from?
- Why does searching the list carry no cost?
- For a proper interpretation a non-trivial amount of syntactic/semantic computation will still have to be carried out (DP_1_ will have to be interpreted as a generalized quantifier, VP will have to be interpreted as a property that is a member of the GQ), and functional material will have to be left out of the computation).
- Why is there no cost involved in the clash between the result of *Agent first* and the functional material that is present?
- How does *Agent first* apply in the case of subject experiencing verbs (hate, admire), or unaccusatives, whose subjects are not agents?

It seems then that the purported simplicity of *Agent first* is not clear from the steps the processor has to take. Now compare this to the derivation by computation:

1. Retrieve V (θ_1_, θ_2_)
2. Disregard –*ed* and *by*
3. Assign θ_2_ to DP_2_
4. Disregard *was*
5. Apply the predication rule between DP_1_ and VP and assign θ_1_ to DP_1_

None of the further issues arises and, the procedure applies both to agentive and to subject experiencer verbs, without unwarranted outcomes for other verb classes.

^5^Note, that it is crucial to distinguish between an expression and its value—for instance between the expression *John* and the individual John denoted by it. This distinction is often overlooked in the psycholinguistic literature, although clearly crucial if one wants to relate processing effects to the working of the memory system.

^6^One might think that such a two-route model is “uneconomical.” However, clearly, each of these routes has its own independent motivation, and given their nature partial overlap between their effects is unavoidable.

^7^For expository purposes we will ignore the subsequent modifications and explanations of Condition B in Reinhart and Reuland (1993); Reuland (2001, 2011a).

^8^

Rule I: Intrasentential Coreference (Grodzinsky and Reinhart, 1993):
NP A cannot corefer with NP B if replacing A with C, C a variable
*A*-bound by B, yields an indistinguishable interpretation.

^9^This analysis entails that locality is not an *intrinsic* property of *himself* qua being an anaphor. In fact, in positions from which *self* cannot move, there is no locality effect. This is what explains the fact that in (i), where *herself* is contained in a coordinate structure from which movement is prohibited there is no locality effect and *herself* can be bound by *Alice* (Reinhart and Reuland, 1991; Reuland, 2011a) despite the latter's distance:

(i) *Alice* was happy that the King invited the Rabbit and *herself* for tea

^10^This necessitates a rethinking of the conception of binding in the experimental literature, where it is mostly assumed that binding is just a matter of the anaphor looking for an antecedent.

^11^A simple illustration is provided by 1^st^ person plural pronouns in Brazilian Portuguese. It has two forms: *nós*, which is both formally and semantically 1^st^ person plural, and *a gente*, which is formally 3^rd^ person singular, but semantically 1^st^ person plural. *Nós* is free to semantically bind *a gente* and vice versa, but not when they are too close. In that case Agree causes a syntactic feature clash. But crucially, this clash cannot be bypassed by immediately going to the semantics. Note that this exposition is highly simplified. See Reuland (2011a) for the details.

^12^As brought up by one of the reviewers, since Chien and Wexler (1991) and Grodzinsky and Reinhart (1993) there has been considerable discussion about the status of the Delay of Principle B effect. For this discussion two issues must be distinguished. First, there is the question of whether there is a delay in the proper interpretations of pronominals at all. Second, as argued by both Chien and Wexler, and Grodzinsky and Reinhart, the delay shows up primarily with referential antecedents and not with quantificational antecedents. Elbourne (2005) expresses concerns about the adequacy of the experimental designs in these and subsequent studies that argue for such a difference between referential and quantificational antecedents. Conroy et al. (2009) present a number of new experiments that also call this contrast into question, coupled with an extensive overview of experiments discussed in the literature. Summarizing these contributions, it is clear that more factors have to be controlled for than previously assumed, not only involving the design but also the morpho-syntactic composition of the pronominal elements being studied (e.g., Baauw, 2002; Hartman et al., unpublished manuscript). However, even so, one can still maintain that children are more susceptible to Principle B violations than adults. Moreover, none of the literature cited calls into question that children behave quite adult-like with respect to condition A. Consequently, the general claim in the main text is not at issue.

^13^Note that such a general least effort principle is highly implausible on other grounds, given the existence of long-distance anaphors in many languages, for instance, in Scandinavian.

^14^We use this term since it is so entrenched in the literature. But note that the “sloppy interpretation” is the one that does require grammatical operations. So, this is the one that is not shallow.

^15^One might wonder if perhaps even a simpler mechanism might work, namely a preference for an antecedent that is as local as possible. This, however, would not derive the parallelism the construction shows. One of the available options is a “3rd party” reading, as for instance in *John* loves his_1_ *cat and Peter does love his*_2_ *cat too*, where *his*_1_ could be Charles given a suitable context. If *his*_1_ is Charles, *his*_2_ has to be as well. This shows that there is a dependency between the two occurrences of *his* that has to be represented in the licensing mechanism.

^17^Frazier and Clifton also discuss another empirical puzzle, based on their experiment 1a, a self-paced reading experiment. In this experiment they compare VP ellipsis internal to a sentence with VP ellipsis across sentences. Sentences (a) and (b) are neutral in the sense that they are easily compatible both with a BV and a COR interpretation, whereas (c) and (d) are biased in favor of a COR interpretation.

a. John thinks it's a good idea to shave himself before he goes to sleep and Andy does too. (Bound/Neutral, one sentence)
b. John thinks it's a good idea to shave himself before he goes to sleep. Andy does too. (Bound/Neutral, two sentences)
c. John thinks it's a good idea to shave himself before he goes to sleep and Anne does too. (Coreferential, one sentence)
d. John thinks it's a good idea to shave himself before he goes to sleep. Anne does too. (Coreferential, two sentences)

The puzzle this experiment raises is that the BV advantage seems to disappear across a sentence boundary as in the (b) and (d) cases. If so, this would suggest that whatever one sees in VP ellipsis is not the manifestation of a unified phenomenon. As already noted, the interpretation of their results is not entirely clear-cut due to the limitations of their design. In this case another complication arises. In the contrast between these sentence types three factors are involved: i. Reflexivization of shave by himself; ii. Control: assigning a value to PRO in PRO to shave himself; iii. The interpretation of the implicit argument of good idea for x (PRO to shave himself) as either Andy, or John (assuming Anne to be ruled out due to the feature mismatch with himself). The latter constitutes a crucial independent factor, which should have been controlled for, in order for a proper interpretation of this result to be possible. The experiment, then, appears to bear on the interpretation of implicit arguments, rather than on VP ellipsis and the LF-first hypothesis directly.

^18^Note that these results confirmed the findings of the self-paced reading experiments reported by Frazier and Clifton (2000). Hence, across methodologies and languages there is evidence that readers prefer to assign a sloppy identity to ambiguous elliptic structures.

^19^The interpretation of their Experiment 2 is not entirely straightforward due to the presence of two c-commanding potential antecedents, but the findings of this experiment seem to be consistent with the predictions of the PoB model.

^20^Quite interestingly, many of the facts discussed in Karimi and Ferreira (2015) are consistent with the idea that there is a cost associated with accessing discourse. The essence of shallow-R processing of anaphoric dependencies appears to consist of foregoing or postponing the access to discourse, leaving pronominals unvalued.

^21^Many further interesting issues about the processing of interpretive dependencies arise. One factor that sets the processing of pronominals apart from the processing of SELF-anaphors, is that pronominals don't have to be bound, whereas SELF-anaphors in non-exempt positions and simplex anaphors must be bound. In argument positions SELF-anaphors and bound pronominals are in complementary distribution, but not in locative and directional PPs. It would be interesting to investigate the effect of such non-complementarity. Also non-local binding of simplex anaphors in Dutch, German, and Mainland Scandinavian languages raises interesting issues. They must be bound within the sentence – although their domain varies. Especially in Scandinavian they allow a choice of antecedents, and in the non-local domain they are not in complementary distribution with bound pronominals. The question is, then, how precisely these factors show up in the processing of these elements.
